# The assessment of clinical competence in medical students during the COVID-19 pandemic: a scoping review

**DOI:** 10.1007/s11845-026-04431-x

**Published:** 2026-05-04

**Authors:** Harry McGrath, Elysha Brennan, Dominic Harmon

**Affiliations:** 1 University Hospital Limerick, Dooradoyle, Limerick, Ireland; 2 Crumlin Hospital, Children’s Health Ireland, Crumlin, Dublin, Ireland

**Keywords:** COVID-19, Medical education, Clinical competence, OSCE, Assessment validity, Scoping review, Medical students

## Abstract

**Background:**

The COVID-19 pandemic necessitated unprecedented changes to clinical education and assessment. Restrictions on in-person clinical encounters led to rapid adoption of digital assessment modalities, raising concerns about graduate preparedness and the validity of pandemic-adapted assessment approaches. Wojniusz, [40].

**Objectives:**

This scoping review aimed to: (1) map approaches to assessing clinical competence in medical students during COVID-19; and (2) evaluate the validity, reliability, feasibility, and acceptability of pandemic-adapted assessments.

**Methods:**

Following Arksey and O’Malley’s framework, we systematically searched six databases (PubMed/MEDLINE, Scopus, Web of Science, EMBASE, ERIC, Google Scholar) for peer-reviewed empirical studies published March 2020-March 2023 examining clinical competence assessment in final-year medical students. Studies were analyzed using Van der Vleuten’s utility framework (validity, reliability, feasibility, acceptability) and Miller’s Pyramid to categorize competency levels assessed.

**Results:**

From 1,247 references, 13 studies met inclusion criteria, representing 8 countries. Virtual OSCEs were the predominant assessment method (*n* = 8, 62%). Study designs were predominantly weak: surveys (*n* = 6), evaluative case studies (*n* = 4), and observational studies (*n* = 2), with only three cohort studies providing stronger evidence. Most studies (92%) focused on feasibility and acceptability rather than validity or psychometric properties. Physical examination and procedural skills assessment remained a critical unresolved limitation across all digital modalities. No studies employed generalizability theory or Kane’s validity framework.

**Conclusions:**

A paucity of rigorous research on pandemic-adapted assessment validity exists. Published studies employed weak designs producing non-generalizable findings focused on feasibility over validity. Urgent research priorities include: longitudinal cohort studies comparing pandemic-trained graduates to pre-pandemic cohorts; psychometric validation of hybrid assessment models now institutionalized; application of contemporary validity frameworks to digital assessments; and equity analysis of differential impacts across student populations. The field requires systematic validity evidence before permanently adopting pandemic-adapted approaches.

## Introduction

The COVID-19 pandemic necessitated unprecedented transformations in medical education, disrupting traditional approaches to clinical teaching and assessment worldwide [14, 31]. Social distancing requirements precluded in-person clinical placements and objective structured clinical examinations (OSCEs), forcing rapid adoption of virtual learning environments and digital assessment modalities [2, 8]. While this pivot enabled educational continuity during crisis conditions, it raised fundamental questions about whether pandemic-adapted assessments maintained sufficient validity to ensure graduate competence. Smith, [34].

Concerns about graduate preparedness emerged early in the pandemic. Medical students expressed apprehension that reduced clinical exposure might compromise their readiness for practice [7, 21], while educators questioned whether virtual assessments could adequately evaluate clinical skills—particularly physical examination and procedural competencies [13, 18]. These concerns gained urgency as institutions transitioned from emergency remote assessment to more permanent hybrid models, suggesting pandemic adaptations may persist long after social distancing requirements ended [17]. Wojniusz, [40].

From a medical education scholarship perspective, the pandemic created a natural experiment in assessment methodology. Miller’s Pyramid [27] provides a foundational framework for understanding these changes: COVID-19 disproportionately disrupted “shows how” and “does” levels—requiring direct observation of clinical performance—while “knows” and “knows how” cognitive assessments transitioned more seamlessly to digital formats. Contemporary validity theory, particularly Kane’s framework emphasizing scoring, generalization, extrapolation, and implications [10, 20], offers additional lens for evaluating whether pandemic-adapted assessments maintained validity across these inference chains.

Despite substantial literature describing institutional adaptations, remarkably little research has systematically examined the psychometric properties and validity evidence for pandemic-adapted assessments. Existing reviews have either focused broadly on medical education adaptations without specific attention to assessment [11, 14], examined feasibility without validity [22], or concentrated on specific assessment types [6] rather than comprehensive evaluation across modalities. This gap is concerning given that assessment drives learning [38] and determines fitness for practice—any validity compromise potentially impacts patient safety.

Recent umbrella reviews confirm the evidence base remains problematically weak: Nejadghaderi et al., [29] found 96.4% of systematic reviews on COVID-19 medical education rated “critically low” quality using AMSTAR-2 criteria, with assessment receiving only 8.7% of research attention despite its critical importance [11]. This scoping review addresses these gaps by systematically mapping pandemic-adapted assessment approaches in final-year medical students and critically analyzing their reported validity, reliability, feasibility, and acceptability using Van der Vleuten’s utility framework [37].

## Research objectives

This scoping review aims to:

1. Describe approaches to assessing clinical competence in medical students during COVID-19 (March 2020-March 2023).
2. Evaluate the validity, reliability, feasibility, and acceptability evidence reported for pandemic-adapted assessments.
3. Identify research gaps and future priorities for assessment validation in post-pandemic medical education.

## Methods

This scoping review followed the five-stage framework established by Arksey and O’Malley [3], enhanced by methodological refinements from Levac et al., [24] and aligned with PRISMA-ScR reporting guidelines [36]. A scoping review design was selected as appropriate for mapping diverse literature, identifying research gaps, and examining evidence breadth rather than effect sizes—consistent with our objective to understand the extent and nature of assessment validity research during the pandemic.

### Stage 1: identifying the research question

Following extensive review of commentary literature on pandemic assessment adaptations, we identified a critical gap: while numerous papers described institutional responses, few examined the psychometric rigor underlying these changes. Our research questions emerged from this gap:

- What approaches to clinical competence assessment were employed for medical students during COVID-19?
- What validity, reliability, feasibility, and acceptability evidence was reported for these assessments?
- What levels of Miller’s Pyramid could be assessed through pandemic-adapted modalities?

### Stage 2: identifying relevant studies

We developed a comprehensive search strategy in consultation with a medical education specialist and information scientist. Six bibliographic databases were systematically searched: PubMed/MEDLINE, Scopus, Web of Science, EMBASE, ERIC (EBSCO), and Google Scholar. The search combined three concept strings using Boolean operators (Table 1):

**Table 1:** Note: This data is mandatory

Search String	Terms
String 1	Medical Assessment OR COVID-19 OR Clinical Skills
String 2	OSCE OR COVID-19 OR Assessment
String 3	Medical Students OR COVID-19 OR Clinical Assessment
Final String	S1 AND S2 AND S3

The search was limited to peer-reviewed, full-text articles published in English from March 2020 to March 2023. This timeframe captured the full pandemic period from initial social distancing implementation through immediate aftermath, accounting for publication lag in empirical research. Database searches were completed in April 2023.

### Stage 3: study selection

Inclusion criteria targeted empirical research examining clinical competence assessment in final-year medical students during COVID-19. We included diverse study designs (cohort, cross-sectional, observational, case studies) to capture the full scope of pandemic adaptations. Inclusion and exclusion criteria are summarized in Table 2. Exclusion criteria eliminated opinion pieces, narrative reviews, studies of health professions other than medicine, preclinical education, and graduate medical education.

**Table 2:** Inclusion/Exclusion Criteria

Inclusion Criteria	Exclusion Criteria
Undergraduate Medical Education in the final two years prior to graduation.	1. Graduate medical education. 2. Education of health professions other than medicine. 3. Studies of clinical competence in preclinical years.
Publications (Mar 2020 – Mar 2023)	Clinical Assessment Publications prior to March 2020
The assessment of clinical competence with a focus on the assessment of clinical skills and procedural skills	The assessment of clinical knowledge
Empirical studies of the assessment of clinical competence	Opinion pieces, narrative views on the assessment of clinical competence.
Publications in English	Publications in languages other than English.

Two reviewers independently screened titles and abstracts, with conflicts resolved through discussion. Full-text screening of potentially relevant articles followed the same process. Inter-rater reliability was assessed at both stages, achieving substantial agreement (κ = 0.82 for title/abstract screening; κ = 0.89 for full-text screening).

### Stage 4: charting the data

A standardized data extraction form was developed in Microsoft Excel, pilot-tested on three studies, and refined. Extracted data included: author, year, country, study design, sample size, assessment type, delivery platform, Miller’s Pyramid level, validity evidence, reliability measures, feasibility outcomes, acceptability measures, and limitations. Each included study was read multiple times before extraction to ensure comprehension of methodological nuances.

### Stage 5: collating, summarizing, and reporting

We analyzed included studies using Van der Vleuten’s utility framework [37], which conceptualizes assessment utility as the product of validity × reliability × feasibility × acceptability × cost. Miller’s Pyramid [27] provided an additional organizing framework for categorizing competency levels assessed. Given substantial heterogeneity in study designs and outcomes, narrative synthesis was employed rather than meta-analysis. Studies were categorized by design quality, assessment modality, and evidence type reported, with frequency counts and thematic patterns identified.

## Results

### Study selection and characteristics

**Fig. 1:**
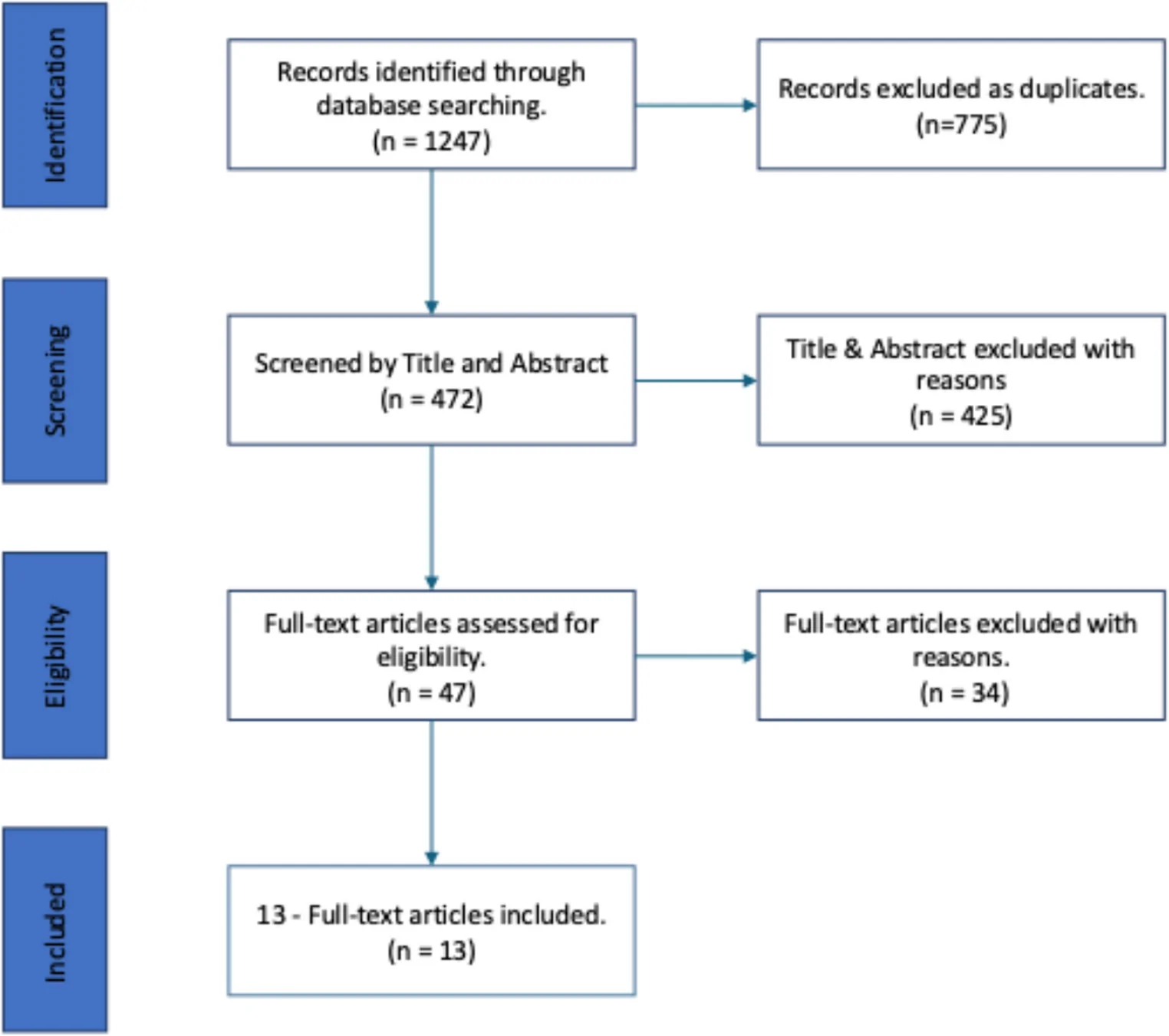
PRISMA Diagram illustrating the search and select process

The database searches identified 1,247 references. After removing duplicates (*n* = 312), 935 references underwent title/abstract screening. Of these, 89 proceeded to full-text review. Ultimately, 13 studies met all inclusion criteria. Key characteristics of included studies are summarized in Table 3 and were included in the scoping review. The main exclusion reasons at full-text stage were: wrong population (*n* = 31, primarily graduate medical education), wrong outcome (*n* = 24, knowledge assessment only), and study design (*n* = 21, opinion pieces or narrative reviews). Figure 1 presents the PRISMA flow diagram.

**Table 3:** Study Characteristics

Year of Publication			Type of Assessment		
2020.		1	Virtual OSCE-	8	
2021.		6	VCEE		1
2022.		6	Simulation		1
			Not stated		3
Country of Origin			* VCEE (Virtual Clinical Encounter Examination)		
Australia.		1			
Brazil.		1			
Canada	1		Research Design		
Egypt.		1	Cohort Study		3
Germany		1	Survey design		6
UAE.	2		Comparative		1
UK.		4	Descriptive		1
United States (USA).	2		Observational		2
Assessment delivery medium					
LMS		2			
Web conferencing		7			
Not defined		4			

Included studies represented 8 countries across 4 continents: United Kingdom (*n* = 4), United Arab Emirates (*n* = 2), United States (*n* = 2), Australia (*n* = 1), Brazil (*n* = 1), Canada (*n* = 1), Egypt (*n* = 1), and Germany (*n* = 1). Publication years were: 2020 (*n* = 1), 2021 (*n* = 6), 2022 (*n* = 6). The predominance of 2021–2022 publications reflects both pandemic timing and typical publication lag for empirical research.

### Study designs and methodological quality

Study designs varied substantially, with most employing relatively weak methodologies:

- Cross-sectional surveys: *n* = 6 (46%).
- Evaluative case studies: *n* = 4 (31%).
- Cohort studies: *n* = 2 (15%).
- Comparative studies: *n* = 1 (8%).

The predominance of surveys and case studies limits generalizability of findings. Only three studies employed designs capable of producing generalizable evidence (two cohort studies, one comparative study with historical controls). Sample sizes ranged from 9 to 160 participants (median = 61), with four studies not reporting sample sizes at all.

Critically, no studies employed contemporary psychometric approaches such as generalizability theory, item response theory, or Kane’s validity framework. Standard setting procedures were rarely described. This represents a substantial methodological gap given the high-stakes nature of clinical competence assessment.

Assessment Modalities and Delivery Platforms.

Detailed extracted data for included studies are presented in Table 4.

**Table 4:** Data Extraction Table

Title	Authors	Year	Participants	Design	Findings
Misjudgment of Skills in Clinical Examination Increases in Medical Students Due to a Shift to Exclusively Online Studies during the COVID-19 Pandemic.	Lechner er al	2022	31	Correlational comparative study	This study revealed that medical students with online-only training during the COVID-19 pandemic regulations achieved similar ENT examination skills as those with a traditional on-campus training before the pandemic.
Assessing the utility and efficacy of e-OSCE among undergraduate medical students during the COVID-19 Pandemic.	Shorbagi et al.	2022	105 (3 × 35)	Survey	The aim of this paper is to evaluate the utility and efficacy of an electronic Objective Structured Clinical Examination (e-OSCE), which attempted to transform the format of a face-to-face OSCE to an e-OSCE.93.4%of students and 84.3% examiners agreed with the quality and process of e-OSCE.
Assessing Clinical Skills Via Telehealth Objective Standardized Clinical Examination: Feasibility, Acceptability, Comparability, and Educational.	Farrell et al.	2022	160	Survey	It was feasible to adapt a standardized OSCE to a telehealth format when in-person clinical skills assessment was impossible.
The virtual Clinical Assessment of Skills and Competence: The impact and challenges of a digitised final examination.	Chu et al.	2021	N/A	Evaluative case study	Royal College of Psychiatrists delivering the Clinical Assessment of Skills and Competence virtually. This article aims to explore the fairness of such an examination, the difference in trainee experience, and the use of telemedicine to consider what might be lost.
COVID-19 and medical education: Rethinking student assessment- The Virtual Observational Standard Clinical Examination.	Abal et al.	2021	25	Survey	It is feasible to design new ways to assess medical students via online means. B) The experience of an OSCE can be translated to an online environment with minimum technological requirements.
Medical Student Assessment in the Time of COVID-19.	Prigoff et al.	2021	19	Comparative	During COVID-19, students used virtual assessment and paper found that an open book exam and a virtually proctored shelf exam was comparable with 3 years of historical data.
Is online objective structured clinical examination teaching an acceptable replacement in post-COVID-19 medical education in the United Kingdom?: a descriptive study.	Motkur et al.	2022	62	Evaluative case study	This study evaluated the students attitude to online OSCe teaching and assessment, the data suggested that students were unwilling to completely replace in-place teaching.
Changes to objective structured clinical examinations (OSCE) at Australian. medical schools in response to the COVID-19 pandemic	Heal et al.	2021	12 medical schools	Survey	All schools changed their OSCEs in 2020 in response to COVID-19. Modifications varied from reducing station numbers and changing delivery methods to removing OSCE and complete assessment re-structuring. Several innovative methods of OSCE delivery were implemented to preserve OSCE validity and reliability whilst balancing feasibility.
Virtual Clinical Encounter Examination (VICEE): A novel approach for assessing medical students’ non-psychomotor clinical competency.	Hamdy et al.	2021	61		This study used an online AI-based virtual patient high fidelity simulation as an alternative tool to assess some aspects of non-psychometric competencies.
A pandemic means rethinking performance-based assessments.	Tavares et al.	2020	N/A	Evaluative case study	Re-thinking assessment following the pandamic
Assessing the utility of virtual OSCE sessions as an educational tool: a national pilot study.	Grover et al.	2022	85	Survey	Most participants in the virtual OSCE sessions reported an improvement in their confidence in history-taking, communication and data interpretation skills.
Evaluation of the Utility of Online Objective Structured Clinical Examination Conducted During the COVID-19 Pandemic.	Arekat et al.	2022	N/A	Survey	Online OSCE might be a good alternative of conventional clinical assessments in times of crises and impossibility of having in-person contact between students, examiners, and patients. An important major drawback is still present in such initiatives, which is the inability to assess students’ physical examination skills.
Undertaking a high stakes virtual OSCE (“VOSCE”) during Covid-19.	Blythe et al.	2021	9	Evaluative case study	This paper address the limitation of assessing some particular types of skills across the remote platform( practical procedures).

Virtual OSCEs were the dominant assessment modality, employed in 8 studies (62%). Other approaches included:

- Virtual Clinical Encounter Examinations (VCEE): *n* = 1.
- Simulation-based assessment: *n* = 1.
- Not clearly specified: *n* = 3.

Delivery platforms included:

- Web conferencing software (Zoom, Microsoft Teams): *n* = 7 (54%).
- Learning Management Systems: *n* = 2 (15%).
- Not defined: *n* = 4 (31%).

The lack of platform specification in 31% of studies hampers replication and implementation guidance. Moreover, studies rarely described technical specifications, security protocols, or contingency plans for technology failures—all critical feasibility considerations.

### Evidence for validity

Validity evidence was strikingly limited across all studies. Using Kane’s validity framework, we examined four inference types:

Scoring inference: Three studies (23%) reported inter-rater reliability statistics, with coefficients ranging from 0.76 to 0.89. Ten studies (77%) provided no reliability evidence despite employing subjective scoring by multiple examiners. No studies reported generalizability coefficients, standard error of measurement, or decision consistency indices.

Generalization inference: No studies examined whether virtual OSCE performance generalized to in-person clinical settings. The single comparative study [30] found equivalence between virtual and historical pre-pandemic examination scores but did not assess whether this represented true competence equivalence versus assessment difficulty matching.

Extrapolation inference: No studies examined relationships between virtual assessment scores and subsequent clinical performance, supervisor ratings, or patient outcomes. This absence of predictive validity evidence represents the most critical gap—we cannot determine whether pandemic-adapted assessments identified truly competent graduates.

Implications inference: No studies examined consequences of pass/fail decisions, including equity impacts across student populations or effects on remediation and progression. Chu, [9] raised concerns about “fairness” of virtual examinations but provided no empirical evidence.

### Evidence for feasibility and acceptability

In stark contrast to the paucity of validity evidence, 12 studies (92%) reported feasibility or acceptability outcomes—typically through post-assessment surveys. Common findings included:

Student acceptability: Student satisfaction ratings were generally high (> 85% positive responses in most studies), although some studies reported reluctance to fully replace in-person teaching with online OSCE formats [28]. However, qualitative feedback revealed concerns about inability to demonstrate physical examination skills, technology anxiety, and perception that virtual assessments were “easier” than traditional OSCEs [23, 33].

Examiner acceptability: Examiner satisfaction was lower than student satisfaction, with 84% positive responses. Examiners expressed concerns about inability to assess physical examination skills, difficulty judging student professionalism through screens, and increased cognitive load from technology management [33].

Implementation feasibility: All studies successfully implemented virtual assessments, demonstrating technical feasibility. However, multiple studies reported substantial logistical challenges including: bandwidth limitations, requirement for students to have private quiet spaces, need for technical support staff, and challenges with standardized patient recruitment and training [1, 12].

A critical limitation identified across all studies: physical examination and procedural skills could not be adequately assessed virtually. This represents a fundamental validity threat—if critical competencies cannot be assessed, overall assessment validity is compromised regardless of other quality indicators.

### Miller’s pyramid analysis

Analysis through Miller’s Pyramid revealed differential pandemic impact across competency levels:

“Knows” (factual knowledge): Readily assessed through written examinations, minimally impacted by pandemic adaptations.

“Knows how” (applied knowledge): History-taking, clinical reasoning, and communication skills were successfully assessed virtually. Studies reported these domains translated well to virtual formats [15, 16].

“Shows how” (performance in simulated settings): Severely compromised for physical examination and procedural skills. While history-taking demonstrations were feasible, physical examination could only be assessed through verbal description rather than observation [4, 5].

“Does” (real-world performance): Could not be assessed during pandemic due to restricted clinical access. No included studies examined workplace-based assessment alternatives.

## Discussion

This scoping review reveals a critical gap between the scale of pandemic-driven assessment changes and the rigor of research examining those changes. From 1,247 references, only 13 studies met inclusion criteria, and these predominantly employed weak designs focused on feasibility rather than validity. This paucity of robust evidence is particularly concerning given that assessment determines fitness for practice and patient safety.

### Principal findings

Limited research volume: With thousands of medical schools worldwide implementing pandemic adaptations, finding only 13 empirical studies specifically examining assessment validity represents a striking evidence gap. This aligns with Nejadghaderi et al.‘s (2024) umbrella review finding that 96.4% of COVID-19 medical education systematic reviews were “critically low” quality, and Daniel et al.‘s (2021) finding that assessment received only 8.7% of research attention despite its critical importance.

Weak study designs: The predominance of surveys (46%) and case studies (31%) severely limits generalizability. While these designs provide useful preliminary insights, they cannot establish whether virtual assessments maintained equivalent validity to traditional approaches. Only one study employed comparative design with historical controls, and none used experimental or quasi-experimental designs.

Feasibility prioritized over validity: 92% of studies reported feasibility or acceptability outcomes, while validity evidence was minimal or absent. This pattern reflects crisis-driven priorities—institutions needed workable solutions quickly and documented implementation success. However, as pandemic-adapted approaches become institutionalized [17], this evidence gap becomes increasingly problematic.

Physical examination skills: unresolved limitation: Every study acknowledged inability to assess physical examination and procedural skills virtually. This represents a fundamental validity threat that cannot be addressed through better technology or training—these competencies require direct observation. The field appears to have accepted this limitation without adequate consideration of implications for patient safety.

### Relationship to existing literature

Our findings align with and extend existing reviews. Cartledge, [6] systematically reviewed clinical examination adaptations but noted “variability in reporting” and called for “common frameworks”—our scoping review confirms these limitations persist. Kunutsor, [22] examined virtual OSCE feasibility but not validity; our review demonstrates this feasibility-over-validity pattern characterizes the broader literature.

Recent 2024 publications provide some encouraging methodological advances. Hara, [17] represent the first generalizability theory analysis of hybrid OSCEs, finding G-coefficients (0.759–0.823) comparable to pre-pandemic traditional OSCEs (0.624–0.782). McGee, [26] conducted the first rigorous meta-analysis, synthesizing 27 studies including 13 RCTs, finding beneficial effects on assessment outcomes (mean difference 1.93, 95% CI 1.22–2.64) though with high heterogeneity (I²=97%). These studies demonstrate methodological progress but also highlight that such rigorous research remains rare [19, 32].

Our findings also connect to broader medical education assessment discourse. The “next era of assessment” paradigm emphasizes consequences, equity, and societal impact [25]—yet our review found zero studies examining equity implications of pandemic adaptations. This absence is particularly concerning given documented digital divide issues and evidence that “small differences in assessed clinical performance amplify to large differences in grades and awards” [41] disproportionately affecting underrepresented students.

### Implications for practice and policy

Medical schools face a critical decision: return entirely to pre-pandemic assessment approaches or permanently adopt hybrid models. However, our review demonstrates insufficient evidence exists to make informed decisions. We offer several recommendations:

1. Systematic validation before permanent adoption: Institutions should not permanently adopt pandemic-adapted assessments without rigorous psychometric validation. At minimum, this requires: (a) generalizability studies examining reliability across raters, cases, and occasions; (b) correlation studies with workplace performance; (c) longitudinal tracking of pandemic-trained graduates’ clinical competence; and (d) equity analysis examining differential impacts across student populations.
2. Compensatory assessment strategies: For institutions maintaining virtual assessment components, programmatic assessment approaches may mitigate individual assessment limitations. This requires aggregating evidence across multiple methods, contexts, and time points—reducing reliance on any single high-stakes examination [38]. However, even programmatic approaches require validity evidence for individual components.
3. Faculty development in assessment literacy: The paucity of validity evidence partly reflects limited assessment expertise. Institutions should invest in faculty development covering contemporary validity frameworks [10, 20], standard setting procedures, psychometric analysis, and equity-informed assessment design.
4. Physical examination skills: direct observation essential: Virtual assessment of physical examination skills is not feasible with current technology. Institutions must ensure graduates receive adequate in-person assessment of these critical competencies, possibly through: intensive assessment boot camps, extended clinical rotations with embedded assessment, or simulation-based examinations for high-risk procedures, which have been shown to support clinical skills development during pandemic conditions [39].

### Research priorities

This review identifies several urgent research priorities:

Longitudinal cohort studies: Compare clinical performance, supervisor ratings, and patient outcomes for pandemic-trained graduates versus pre-pandemic cohorts. Follow-up periods should extend at least 2–3 years into practice to detect competency differences that may not manifest immediately.

Psychometric validation of hybrid assessments: Apply generalizability theory, item response theory, and Kane’s validity framework to hybrid OSCE models now being institutionalized. Hara, [17] provide an exemplar, but single-site studies cannot establish generalizability.

Equity and fairness studies: Examine whether pandemic adaptations disproportionately advantaged or disadvantaged particular student groups. Analyze by socioeconomic status, race/ethnicity, international versus domestic students, and disability status. Digital divide issues likely created systematic inequities requiring investigation.

Technology-enhanced physical examination assessment: Explore emerging technologies (haptic feedback systems, augmented reality, AI-enabled motion capture) for assessing physical examination skills remotely. While current technology is inadequate, investment in innovation could address this critical limitation.

Multi-institutional validation studies: Single-institution case studies dominate current literature. Multi-institutional studies with standardized protocols, shared psychometric analysis plans, and coordinated data collection would dramatically strengthen evidence quality [35].

### Strengths and limitations

This review’s strengths include comprehensive search across six databases, dual independent screening, alignment with PRISMA-ScR guidelines, and application of established theoretical frameworks (Miller’s Pyramid, Van der Vleuten’s utility framework, Kane’s validity framework) for analysis. We included diverse study designs to map the full landscape rather than imposing restrictive quality criteria.

Limitations include restriction to English-language publications, which may have excluded relevant research from non-English-speaking countries. We focused specifically on final-year medical students, which provided clear population boundaries but excluded potentially relevant research on graduate medical education or other health professions. The scoping review methodology prioritized breadth over depth; systematic review with quality appraisal would provide different insights.

Importantly, our March 2023 search cutoff means we captured early pandemic literature but missed some recent methodological advances. The Hara et al., [17] and McGee et al., [26] studies published subsequently represent exactly the rigorous research this review calls for—their emergence suggests the field may be transitioning from crisis response to systematic validation.

## Conclusions

The COVID-19 pandemic necessitated rapid, unprecedented changes to clinical competence assessment in medical education. While institutions successfully implemented virtual assessment alternatives—demonstrating impressive agility and innovation—the research base examining validity of these changes remains critically limited. Only 13 studies met inclusion criteria from 1,247 references, and these predominantly employed weak designs focused on feasibility rather than validity.

This evidence gap is particularly concerning as institutions transition pandemic-adapted approaches from emergency measures to permanent practice. Without robust validity evidence, we cannot ensure graduates possess clinical competencies necessary for safe patient care. Physical examination and procedural skills—fundamental to clinical practice—remain fundamentally unassessable through current virtual modalities.

The field requires urgent investment in rigorous validation research employing longitudinal designs, contemporary psychometric approaches, and equity analysis. Until such evidence exists, institutions should approach permanent adoption of pandemic-adapted assessments with appropriate caution, ensuring robust in-person assessment of competencies that cannot be virtually evaluated. Medical education must balance innovation and tradition, but patient safety requires we never compromise assessment validity in pursuit of operational efficiency.

## Data Availability

Not applicable. All data used in this scoping review were extracted from publicly available, peer-reviewed publications identified through database searches. No new datasets were generated.
